# Ethnicity Strongly Influences Body Fat Distribution Determining Serum Adipokine Profile and Metabolic Derangement in Childhood Obesity

**DOI:** 10.3389/fped.2020.551103

**Published:** 2020-10-09

**Authors:** Gabriel Á. Martos-Moreno, Julián Martínez-Villanueva, Rocío González-Leal, Vicente Barrios, Sara Sirvent, Federico Hawkins, Julie A. Chowen, Jesús Argente

**Affiliations:** ^1^Departments of Pediatrics and Pediatric Endocrinology, Hospital Infantil Universitario Niño Jesús, Madrid, Spain; ^2^Department of Pediatrics, Universidad Autónoma de Madrid, Madrid, Spain; ^3^Centro de Investigación Biomédica en Red Fisiopatología Obesidad y Nutrición (CIBEROBN), Instituto de Salud Carlos III, Madrid, Spain; ^4^Department of Radiology, Hospital Infantil Universitario Niño Jesús, Madrid, Spain; ^5^Department of Endocrinology, Hospital Universitario Doce de Octubre, Universidad Complutense de Madrid, Madrid, Spain; ^6^Instituto Madrileño de Estudios Avanzados (IMDEA) Food Institute, Madrid, Spain

**Keywords:** childhood obesity, body fat distribution, DXA-scan, abdominal magnetic resonance, adipokines, insulin resistance, ethnicity, steatosis

## Abstract

**Background:** Body fat content and distribution in childhood is influenced by sex and puberty, but interethnic differences in the percentage and distribution of body fat also exist. The abdominal visceral/subcutaneous fat ratio has been the main feature of body fat distribution found to associate with the serum adipokine profile and metabolic derangement in adulthood obesity. This has also been assumed for childhood obesity despite the known singularities of this disease in the pediatric age in comparison to adults.

**Objective:** We aimed to investigate the effect of ethnicity, together with sex and pubertal status, on body fat content and distribution, serum adipokine profile, metabolic impairment and liver steatosis in children and adolescents with obesity.

**Patients and Methods:** One hundred and fifty children with obesity (50% Caucasians/50% Latinos; 50% males/50% females) were studied. Body fat content and distribution were studied by whole body DXA-scan and abdominal magnetic resonance, and their relationships with liver steatosis (as determined by ultrasonography), glycemia, insulinemia, lipid metabolism, uric acid, total and HMW-adiponectin, leptin, leptin-receptor, and sex steroid levels were explored.

**Results:** Latino patients had more severe truncal obesity (higher trunk/lower limb fat ratio, odds ratio 10.00; *p* < 0.05) and higher prevalence of liver steatosis than Caucasians regardless of sex or pubertal status, but there were no difference in the visceral/subcutaneous abdominal fat ratio, except for pubertal females. A higher trunk/lower limb fat ratio, but not the visceral/subcutaneous abdominal fat ratio, was associated with adipokine profile impairment (higher free leptin index and lower adiponectin levels), insulin resistance and dyslipidemia, and was further enhanced when liver steatosis was present (*p* < 0.05). A higher abdominal visceral/subcutaneous fat ratio was observed in prepubertal children (*p* < 0.01), except for Latino females, whereas predominant subcutaneous fat deposition was observed in adolescents.

**Conclusion:** Ethnicity is one of the main determinants of increased trunk body fat accumulation in Latino children with obesity, which is best estimated by the trunk/lower limb fat ratio and related to the development of metabolic derangement and liver steatosis.

## Introduction

In the last decades, a substantial increase in the immigration rate has occurred in the most populated European countries (150,000 to over 10,00,000 immigrants/year [Eurostat 2016]), with a significant increase in the proportion of migrant children and adolescents seen in the pediatric clinics in our environment. The origins of migrant subpopulations vary widely within European countries [e.g., in Spain there is a predominant Latino (Peruvian, Ecuadorian and Bolivian) ethnic background among immigrants, as do their socioeconomic conditions and health status. This increase in immigration rate, in addition to an often-higher birth rate in immigrants than in local population as well as an increased risk of being overweight determined by migration (1), has substantially increased the diversity of ethnic groups followed in our pediatric obesity clinics.

Profound changes in body composition and proportion occur during growth and pubertal development (2) in a sex-dependent fashion due to the influence of hormones, particularly growth hormone and sex steroids (3). In this evolving scenario, additional changes in body development and proportions are observed when obesity develops affecting body fat content and distribution (4), with postulated ethnic differences because of different genetic and epigenetic backgrounds (5). Children from different ethnic groups have significant differences in body fat and lean mass content for a given BMI, particularly when comparing Latino and European adolescents, which leads to an ethnic-associated variability in the metabolic profile related to obesity (5). The estimation and comparison of trunk and limb adiposity by skin fold measurements indicate that the anatomical distribution of body fat is also influenced by ethnicity (5). Thus, these differences should be taken into consideration in a clinical setting.

The development of more precise methods, such as dual energy X-ray absorption (DXA) scan, abdominal computerized tomography (CT) scan, and magnetic resonance imaging (MRI) (6, 7) has allowed for a more detailed study of the differences in adipose content and distribution and this has been employed in children, especially in the study of obesity, including in accordance to ethnicity, sex and puberty (2). These changes are more evident as age progresses and differences between sexes are highly influenced by pubertal development and growth, resulting in changes regarding central vs. peripheral and visceral vs. abdominal fat distribution (2, 8). Inter-ethnic differences in depot-specific fat accumulation in childhood and adolescent obesity have been described (9), but the influence of sex and puberty on body fat distribution is also reported to differ between different ethnicities (10).

Ectopic fat accumulation in the liver (non-alcoholic fatty liver disease or steatosis) is more highly correlated to insulin resistance than to the total amount or distribution of body fat (11) and highly influenced by ethnicity (12–14) and nutritional habits (15). Nevertheless, it is currently uncertain whether non-alcoholic fatty liver disease may directly cause insulin resistance or vice versa. Therefore, further research is still necessary to clarify this issue (16).

Conversely, the amount of limb fat has been shown to be inversely correlated with insulin resistance (17). However, the physiological evolution and factors influencing the relative amount of trunk/limb or abdominal visceral/subcutaneous fat in children and adolescents according to sex and ethnicity, as well as their modifications in obesity and the pathophysiological relevance, remain insufficiently characterized to date (2, 18). Moreover, the Latin American immigrant populations differ between countries, with Spain having a high incidence of Peruvian, Bolivian and Ecuadorian immigrants, compared to for example the large Mexican immigrant population in the United States and this may also influence outcomes.

Obesity results in a functional change in the endocrine roles of adipose tissue that is influenced by, among other factors, the amount and distribution of body fat accumulation and adipocyte triglyceride content impairing insulin sensitivity (19, 20). However, the circulating adipokine profile in children with obesity is different from that observed in adults with obesity (21, 22), at least in part due to the greater plasticity of adipose tissue during childhood. Therefore, the age at the beginning of obesity, as well as its severity, influence the structure and functioning of adipose tissue (19). The circulating adipokine profile also changes during physiological pubertal development in a sex-dependent fashion in healthy lean children and adolescents (23). However, the cumulative effect of concomitant development of puberty and obesity on the serum adipokine profile and obesity related complications has been lesser explored.

We hypothesized that ethnic background determines differences in whole body and abdominal body fat distribution between Caucasian and a specific population of Latino children and adolescents with obesity, under the influence of sex and puberty and this associated with their serum adipokine profile and degree of metabolic impairment. Thus, the aims of this study were: (1) To explore the existing relationships between ethnic background and whole body (DXA) and abdominal (MRI) body fat distribution, serum adipokine profile and lipid and carbohydrate impairment in children and adolescents with obesity and their interplay with pubertal development and sex and (2) To study the association between ethnic background and liver steatosis in children and adolescents with obesity according to their pubertal status, their body fat distribution and metabolic profile.

## Patients and Methods

### Patients

One hundred and fifty patients with obesity [BMI > +2 SDS both for national and IOTF references (24, 25)] consulting at the obesity clinic in our hospital were studied. The distribution was 75 Caucasians/75 Latinos (primarily Peruvian, Bolivian and Ecuadorian and all living in Spain since birth), 75 males/75 females and 50 pubertal/25 prepubertal patients in each group.

Parental obesity rate was recorded and compared, showing no differences between ethnic groups (Latinos: 41.9% no parental obesity, 40.3% one parent obese, 17.7% both parents obese vs. Caucasians: 50.0, 33.9, and 16.1%, respectively; χ^2^ 0.834, *p* = 0.659).

All patients and their parents or guardians gave informed consent as required by the local ethics committee (Comité Ético y de Investigación con medicamenteos [CEIM]), which had previously approved the study in accordance with the “Ethical Principles for Medical Research Involving Human Subjects” adopted in the Declaration of Helsinki by the World Medical Association (64th WMA General Assembly, Fortaleza, Brazil, October 2013).

### Methods

Weight, height, and BMI were recorded and standardized (23, 24) and an oral glucose tolerance test (OGTT, 1.75 g/kg, maximum 75 g) for glucose and insulin measurements was performed in all patients. Serum was stored at −80°C until assayed. Fasting samples were also used to determine HbA1c, lipid profile and uric acid levels by standardized assays, as well as total and HMW-adiponectin, leptin and leptin soluble receptor (sOBr), testosterone, estradiol, androstendione and dehydroepiandrosterone sulfate (DHEA-S) by using commercial ELISAs and radioimmunoassays, as previously reported (21, 23). Pubertal development was established by evaluating gonadal [G, males] and breast [B, females] development, respectively according to Tanner stages by the same physician in every patient.

To exclude secondary causes of obesity in the study cohort, every patient's clinical record was reviewed, and the patients underwent a full obesity-oriented clinical examination by the same physician in all cases and complementary tests to rule out underlying pathologies (including thyroid function and cortisol production assessment) were performed. Additionally, no patient was found to exhibit pathologic findings in analysis by MS-MLPA for the detection of imprinting disorders, studies of CNVs (copy number variant) or in the sequencing of a panel of 15 obesity candidate genes (26–28).

The free leptin index (FLI), leptin/sOBr and leptin/adiponectin ratio (LEP/ADPr) were calculated. HOMA index was calculated as follows: glucose (mg/dl) x insulin (μU/ml)/405 and WBISI (whole body insulin sensitivity index) as: 10,000/√ fasting glucose x fasting insulin x mean glucose in the OGTT x mean insulin in the OGTT, as previously reported (29, 30).

Patients underwent liver ultrasonography, body composition analyses [DXA (Hologic QDR4500W) and abdominal MRI analyzing images at the umbilical level (1.5T Phillips® Achieva) (31)] for body fat quantification and distribution. Whole body fat content (%) was standardized for age and sex (fat %-SDS) (3). The trunk/tower limb fat ratio (trunk/LL-fat-ratio) and the whole body fat/lean mass ratio (fat/lean-ratio) were calculated in the DXA study and the visceral to subcutaneous abdominal fat ratio (VIS/SQ-fat-ratio) was calculated using MRI data.

Food intake records consisted of a weekly 3-days non-structured self-reported diary and a specific questionnaire of weekly frequency of sweetened beverage consumption. No inter-group differences in the frequency of sweetened beverage consumption was found nor was there a limitation of the access to any specific food group was reported by any family.

### Statistical Analysis

A three-way ANOVA was conducted to study the eventual presence of main effect and/or interaction of the three factors (ethnicity, sex, and puberty) on the analyzed variables, followed by two-way and one-way ANOVA for the study of two factor interaction and single factor main effect on the studied variables, respectively. The F and *p*-values are shown for main factor effects on the studied variables and for the interaction between factors.

Multivariate analysis was performed by using binary logistic regression with the forward conditional method. The dependent variable was ethnic background and independent variables included all variables that were statistically significant in the bivariate analysis or where clinical implication could be plausible. Calibration of the model was performed using the Hosmer-Lemeshow statistic (*p* = 0.496; with C-statistic *p* = 0.856). The discriminatory power was assessed using the area under the ROC curve (receiver-operator characteristics) obtained by analyzing the probability of the value predicted by the multivariate model. The results of the multivariable model were adjusted by sex and puberty and are presented as odds ratio (OR) and its 95% confidence interval.

Student's *t-*test was used for comparison between two independent groups of normally distributed parametric variables, whereas the Mann & Whitney's U test was used for non-normally distributed values. Square Chi test was used for non-parametric variable comparison between groups. All data are reported as the mean ± SD.

The relationships between quantitative normal variables were studied by linear correlation analysis (Spearman's rho was used as most variables did not fit a normal distribution). A value of *p* < 0.05 was chosen as the level of significance.

The software used was Statistical Package for Social Sciences (IBM® SPSS® Statistics for Windows, Version 20.0. IBM Corp. Armonk, NY, USA).

## Results

### Body Composition and Fat Distribution

The study of standardized fat mass percentage (DXA) showed a significant main effect of sex (higher in females, F: 24.7; *p* < 0.001) and puberty (higher in prepubertal children, F: 7.0; *p* < 0.01), but not of ethnicity. No influence of sex, puberty or ethnicity was observed on BMI-SDS or fat/lean ratio (Table 1).

**Table 1:** Demographic, anthropometric, body composition, adipokine, and metabolic characterization and prevalence of liver steatosis according to sex, ethnic background, and pubertal status.

	**Caucasians (*****n*** **=** **75)**		**Latinos (*****n*** **=** **75)**		**Main effect (*p*-value)**	**Interaction (*p*-value)**
	**Prepubertal**	**Pubertal**	**Prepubertal**	**Pubertal**		
*n*	25 (33.3%)	50 (66.7%)	25 (33.3%)	50 (66.7%)		
Gender	F 12 (48%) M 13 (52%)	F 25 (50%) M 25 (50%)	F 11 (45.8%) M 13 (54.2%)	F 26 (51%) M 25 (49%)		
Age (years)	F 8.07 ± 1.62 M 10.06 ± 1.84	F 13.56 ± 2.38 M 13.95 ± 1.79	F 7.79 ± 1.81 M 9.43 ± 1.75	F 12.07 ± 1.85 M 13.50 ± 1.67		
BMI-SDS	F 4.21 ± 1.78 M 5.04 ± 1.63	F 4.09 ± 1.55 M 3.95 ± 1.09	F 3.60 ± 1.09 M 4.33 ± 1.28	F 4.15 ± 1. 36 M 3.74 ± 1.13	N.S.	N.S.
Fat (%)-SDS	F 3.15 ± 0.42 M 3.95 ± 0.80	F 2.78 ± 0.91 M 3.60 ± 0.92	F 2.99 ± 0.59 M 3.61 ± 0.74	F 2.46 ± 0.75 M 3.24 ± 1.05	Puberty (*p* < 0.01) Sex (*p* < 0.001)	N.S.
Fat/Lean	F 0.78 ± 0.07 M 0.73 ± 0.13	F 1.14 ± 1.79 M 0.64 ± 0.16	F 0.75 ± 0.08 M 0.66 ± 0.09	F 0.73 ± 0.14 M 0.60 ± 0.16	N.S.	N.S.
Trunk/Lower Limbs	F 1.28 ± 0.17 M 1.34 ± 0.23	F 1.27 ± 0.23 M 1.23 ± 0.29	F 1.55 ± 0.40 M 1.36 ± 0.18	F 1.43 ± 0.26 M 1.38 ± 0.25	Ethnicity (*p* < 0.01)	N.S.
Visceral/ Subcutaneous	F 0.25 ± 0.07 M 0.24 ± 0.07	F 0.16 ± 0.05 M 0.20 ± 0.05	F 0.24 ± 0.08 M 0.29 ± 0.10	F 0.21 ± 0.07 M 0.21 ± 0.05	Puberty (*p* < 0.001)	Puberty * ethnicity * sex (*p* < 0.05)
Leptin	F 22.13 ± 9.38 M 24.66 ± 10.69	F 39.75 ± 16.03 M 26.46 ± 20.37	F 22.56 ± 10.48 M 18.98 ± 6.11	F 29.74 ± 15.30 M 19.55 ± 13.01	Puberty (*p* < 0.01) Ethnicity (*p* < 0.05) Sex (*p* < 0.05)	Puberty * Sex (*p* < 0.05)
sOBr	F 22.59 ± 7.85 M 20.22 ± 6.35	F 13.29 ± 2.51 M 16.42 ± 5.83	F 19.11 ± 5.51 M 23.30 ± 5.37	F 14.19 ± 4.67 M 17.05 ± 4.71	Puberty (*p* < 0.001) Sex (*p* < 0.05)	N.S.
FLI	F 1.31 ± 1.21 M 1.41 ± 0.92	F 3.29 ± 1.97 M 1.92 ± 1.63	F 1.39 ± 0.97 M 0.85 ± 0.31	F 2.63 ± 2.08 M 1.39 ± 1.63	Puberty (*p* < 0.001) Sex (*p* < 0.01)	N.S.
Adiponectin	F 10.64 ± 3.48 M 9.23 ± 4.42	F 9.82 ± 3.81 M 11.50 ± 12.14	F 12.27 ± 4.24 M 12.39 ± 6.26	F 10.59 ± 3.18 M 9.59 ± 5.34	N.S.	N.S.
HMW-Adiponectin	F 4.95 ± 2.31 M 5.25 ± 3.10	F 5.29 ± 3.20 M 4.45 ± 2.46	F 5.48 ± 1.86 M 5.20 ± 2.77	F 4.93 ± 2.36 M 4.37 ± 3.29	N.S.	N.S.
Leptin/ Adiponectin	F 2.41 ± 1.60 M 4.16 ± 5.60	F 4.72 ± 3.15 M 3.27 ± 2.93	F 2.09 ± 1.22 M 1.83 ± 0.93	F 3.15 ± 2.09 M 2.37 ± 1.53	Ethnicity (*p* < 0.01)	Puberty * Sex (*p* < 0.05)
HOMA	F 2.48 ± 1.46 M 2.44 ± 1.16	F 3.47 ± 1.66 M 3.66 ± 2.06	F 4.68 ± 4.10 M 2.99 ± 1.19	F 4.43 ± 4.18 M 4.01 ± 1.76	Ethnicity (*p* < 0.05)	N.S.
WBISI	F 4.83 ± 3.16 M 5.03 ± 3.60	F 4.12 ± 1.75 M 3.44 ± 1.50	F 3.02 ± 2.33 M 4.24 ± 1.67	F 3.80 ± 2.35 M 3.34 ± 1.55	N.S.	N.S.
LDL-C (mg/dl)	F 98.93 ± 22.50 M 106.86 ± 24.91	F 94.62 ± 22.73 M 93.58 ± 26.16	F 96.78 ± 32.61 M 101.14 ± 22.09	F 86.71 ± 16.14 M 94.71 ± 19.11	Puberty (*p* < 0.05)	N.S.
HDL-C (mg/dl)	F 38.70 ± 9.39 M 51.14 ± 11.09	F 43.28 ± 9.34 M 40.76 ± 7.44	F 39.98 ± 10.76 M 47.17 ± 10.87	F 42.42 ± 8.92 M 40.16 ± 11.73	Sex (*p* < 0.05)	Puberty * Sex (*p* < 0.001)
Triglycerides (mg/dl)	F 63.42 ± 22.89 M 57.15 ± 17.00	F 83.44 ± 66.06 M 71.72 ± 36.45	F 103.18 ± 65.41 M 71.54 ± 40.74	F 85.23 ± 50.70 M 86.92 ± 46.43	Ethnicity (p < 0.05)	N.S.
Uric acid (mg/dl)	F 5.03 ± 1.18 M 5.06 ± 0.84	F 5.16 ± 0.69 M 6.22 ± 1.50	F 4.37 ± 1.29 M 4.35 ± 0.92	F 4.85 ± 1.03 M 5.22 ± 1.25	Puberty (*p* < 0.001) Ethnicity (*p* < 0.001)	N.S.

*Significant main effects and interactions between of the three studied factors (ethnicity, sex, and puberty) on the studied variables are shown. All data are reported as the mean ± SD. C, Cholesterol; F, Female; FLI, Free leptin index; M, Male; NS, Non-significant; SDS, Standard deviation [Z] score; sOBr, Leptin soluble receptor*.

Ethnicity showed a main effect on the trunk to lower limb fat ratio (F: 10.3; *p* < 0.01) with the regression analysis showing an OR 10.00 (p < 0.05) for Latinos, and no effect of puberty or sex (Tables 1, 2).

**Table 2:** Multivariate analysis.

**LATINO**	**odds ratio**	**Standard error**	**Z**	**p**	**95% confidence interval**
Sex	1.01	0.50	0.02	0.985	0.39–2.64
Puberty	1.30	0.66	0.52	0.600	0.48–3.52
Liver steatosis	6.78	4.38	2.96	** < 0.01**	1.91–24.08
Triglycerides	1.01	0.01	2.02	** <0.05**	1.00–1.03
LDL cholesterol	0.98	0.12	−2.03	** <0.05**	0.95–1.00
Uric acid	0.52	0.12	−2.81	** <0.01**	0.33–0.82
WBISI	0.75	0.11	−2.01	** <0.01**	0.57–0.99
Leptin/Adiponectin	0.65	0.01	−2.79	** <0.05**	0.49–0.88
Trunk/Lower-limbs	10.00	9.61	2.40	** <0.05**	1.52–65.74

*Dependent variable: ethnic background (independent variables shown in the first column). Hosmer-Lemeshow statistic (p = 0.496) & C-statistic (p = 0.856). The results of the multivariable model were adjusted by Gender and Puberty and we present odds ratios for LATINO ethnicity and their 95% confidence interval. WBISI, Whole body insulin sensitivity index. Bold values indicate the significant results*.

A main effect of puberty on the VIS/SQ-fat-ratio was observed (higher in prepubertal children, F: 25.5; *p* < 0.001). There was a significant interaction between ethnicity, sex and puberty on the VIS/SQ-fat-ratio (F: 4.1; *p* < 0.05). Female Caucasian adolescents had a significantly lower VIS/SQ-fat-ratio than prepubertal Caucasian females (estimated marginal mean difference −0.086 ± 0.023; *p* < 0.001) and lower than Caucasian pubertal males (estimated marginal mean difference −0.038 ± 0.019; *p* < 0.05). These differences were not found in the female Latino adolescents whose VIS/SQ-fat-ratio was significantly higher compared to Caucasian adolescents (estimated marginal mean difference +0.041 ± 0.019; *p* < 0.05) (Table 1).

Trunk/LL-fat-ratio was the body composition parameter that showed the highest correlation with metabolic parameters and adipokine levels. It was positively correlated with HOMA and triglyceride levels and negatively correlated with HDL, total and HMW-adiponectin levels (Table 3). The fat/lean-ratio positively and visceral abdominal fat % negatively correlated with FLI, whereas the VIS/SQ-fat-ratio directly correlated with uric acid levels (Table 3).

**Table 3:** Correlations between body fat distribution parameters, adipokine and metabolic parameters.

	**FLI**	**Adiponectin**	**HMW-Adiponectin**	**Leptin/** **Adiponectin**	**HOMA**	**WBISI**	**Triglycerides**	**HDL-C**	**Uric acid**
**BMI-SDS**									
rho *p-value*	0.42 <0.001	NS	NS	0.41 <0.001	0.34 <0.001	−0.26 <0.01	NS	NS	−0.17 <0.05
**Fat % – SDS**									
rho *p-value*	0.22 <0.01	NS	NS	0.27 <0.01	NS	NS	NS	NS	0.17 <0.05
**Fat/Lean ratio**									
rho *p-value*	0.24 <0.01	NS	NS	NS	NS	NS	NS	NS	NS
**Trunk/Lower Limb ratio**									
rho *p-value*	NS	−0.20 <0.05	−0.21 <0.05	NS	0.21 <0.05	NS	0.24 <0.01	−0.20 <0.05	NS
**VIS/SQ ratio**									
rho *p-value*	NS	NS	NS	NS	NS	NS	NS	NS	0.23 <0.01
**Visceral fat%**									
rho *p-value*	−0.24 <0.01	NS	NS	NS	NS	NS	NS	NS	NS

*C, Cholesterol; FLI, Free leptin index; NS, Non-significant; SDS, Standard deviation [Z] score; SQ, subcutaneous; VIS, visceral*.

Trunk/LL-fat ratio did not correlate with steroid hormone levels. However, the VIS/SQ-fat-ratio and the percentage of visceral abdominal fat negatively correlated with testosterone, estradiol, adrostendione and DHEA-S in females. Conversely, whole body fat %-SDS and the fat/lean-ratio negatively correlated with testosterone levels in males (Table 4).

**Table 4:** Correlations between sex steroids, body fat distribution parameters and adipokine serum levels in both sexes.

	**VIS/SQ r**	**Visc fat %**	**Fat/LeanR**	**T/LLR**	**Fat % - SDS**	**Leptin**	**sOBr**	**FLI**	**Adiponectin**	**HMW-Adiponectin**	**Leptin/** **Adiponectin**
**TESTOSTERONE**											
M rho *p-value*	NS	NS	−0.42 0.001	NS	−0.33 <0.01	−0.23 <0.05	−0.28 <0.05	NS	NS	NS	NS
F rho *p-value*	−0.58 <0.001	−0.60 <0.001	NS	NS	NS	0.37 <0.01	−0.46 <0.001	0.40 <0.01	−0.23 <0.05	NS	0.30 <0.05
**ESTRADIOL**											
M rho *p-value*	NS	NS	NS	NS	NS	NS	−0.29 <0.05	NS	NS	−0.29 <0.05	NS
F rho *p-value*	−0.37 <0.01	−0.38 <0.01	NS	NS	−0.26 <0.05	0.36 <0.05	−0.27 <0.05	NS	NS	NS	NS
**ANDROSTENDIONE**											
M rho *p-value*	NS	NS	NS	NS	NS	NS	−0.39 <0.01	NS	−0.26 <0.05	NS	NS
F rho *p-value*	−0.53 <0.001	−0.54 <0.001	NS	NS	NS	0.29 <0.05	−0.40 <0.01	0.36 <0.01	−0.33 <0.05	NS	0.31 <0.05
**SDHEA**											
M rho *p-value*	NS	NS	NS	NS	NS	NS	−0.38 <0.01	NS	NS	NS	NS
F rho *p-value*	−0.39 <0.01	−0.41 <0.01	NS	NS	−0.31 <0.05	0.32 <0.05	−0.43 <0.001	0.38 <0.01	−0.32 <0.05	NS	0.38 <0.01

*F, Female; FLI, Free leptin index; LL, Lower lumb; M, Male; NS, Non-significant; R, ratio; SDS, Standard deviation [Z] score; sOBr, leptin soluble receptor; SQ, subcutaneous; T, trunk; VIS, visceral*.

### Metabolic Features and Serum Adipokine Profile

A main effect of ethnicity on HOMA index (higher in Latinos, F: 5.3; *p* < 0.05) and triglyceride levels (higher in Latinos, F: 4.6; *p* < 0.05) was observed (Table 1). The stepwise logistic regression model showed a significant OR below 1 for Latinos for WBISI (OR 0.75; *p* < 0.05) and above 1 for triglycerides (OR1.02; *p* < 0.05), adjusted for age and puberty (Table 2).

There was a main effect of sex on HDL levels (lower in males, F: 4.6; *p* < 0.05) and a significant interaction between sex and puberty with lower HDL in pubertal males (estimated marginal mean difference −8.70 ± 2.40 vs. prepubertal males; *p* < 0.001).

Only puberty showed a main effect on LDL levels in the ANOVA (higher in pubertal children, F: 4.5; *p* < 0.05; Table 1), whereas the stepwise logistic regression model showed a significant OR below 1 for Latinos (OR 0.98; *p* < 0.05; Table 2).

Uric acid levels were influenced by main effects of ethnicity (higher in Caucasians, F: 14.7; p < 0.001 with an OR below 1 in Latinos [OR 0.52; *p* < 0.01]) and puberty [higher in pubertal patients, F: 14.3; *p* < 0.001) (Tables 1, 2).

Leptin and sOB-R were both influenced by sex and puberty, whereas ethnicity showed a main effect only on leptin levels. Leptin levels were significantly higher in females (F: 5.9; *p* < 0.05), Caucasians (F: 4.8; *p* < 0.05) and pubertal patients (F: 7.2 *p* < 0.01), with an interaction between sex and puberty, showing significantly higher levels after puberty only in females (estimated marginal mean difference +12.40 ± 3.45 vs. prepubertal females; *p* < 0.01). On the other hand, sOB-R levels were significantly influenced by puberty (higher in prepubertal children, F: 43.5; *p* < 0.001) and sex (higher in males, F: 4.512; df 1; *p* < 0.05). As a result, FLI was affected by puberty (F: 14.5; *p* < 0.001) and sex (F: 7.3; *p* < 0.01; Table 1).

No main effects of sex, ethnicity or puberty were observed on total or HMW adiponectin or on their ratio. However, a main effect of ethnicity on the LEP/ADP ratio was found (higher in Caucasians; F: 54.0; *p* < 0.01 and OR 0.65 in Latinos in the stepwise logistic regression model [*p* < 0.01]) with an existing interaction between sex and puberty. The LEP/ADP ratio was significantly higher in pubertal compared to prepubertal females (estimated marginal mean difference +1.69 ± 0.68 vs. prepubertal females; *p* < 0.05); Tables 1, 2), but with no effect of pubertal status in males.

### Correlations Between Adipokine, Metabolic Hormone and Steroid Hormone Levels

After controlling for BMI, the Lep/ADP ratio and FLI correlated positively with HOMA and negatively with WBISI. In contrast, total and HMW-adiponectin levels correlated negatively with HOMA and uric acid levels and positively with WBISI and HDL levels. Triglyceride levels were negatively correlated with HMW-adiponectin and positively correlated with LEP/ADPr (Table 5)

**Table 5:** Partial correlations (controlling for the effect of BMI) between serum adipokine and metabolic parameters levels.

**Control variable: BMI-SDS**	**FLI**	**Adiponectin**	**HMW-Adiponectin**	**Leptin/** **Adiponectin**	**HOMA**	**WBISI**	**Triglycerides**	**HDL-C**	**Uric acid**
**FLI**									
rho *p-value*		NS	NS	0.52 <0.001	0.46 <0.001	−0.28 <0.01	NS	NS	NS
**Adiponectin**									
rho *p-value*	NS		0.72 <0.001	−0.48 <0.001	−0.20 <0.05	0.26 <0.01	NS	0.30 <0.01	−0.40 <0.001
**HMW-Adiponectin**									
rho *p-value*	NS	0.72 <0.001		−0.31 <0.001	−0.19 <0.05	0.28 <0.01	−0.20 <0.05	0.24 <0.01	−0.38 <0.001
**Leptin/Adiponectin**									
rho *p-value*	0.52 <0.001	−0.48 <0.001	−0.31 <0.001		0.32 <0.001	−0.28 <0.01	0.20 <0.05	NS	NS
**HOMA**									
rho *p-value*	0.46 <0.001	−0.20 <0.05	−0.19 <0.05	0.32 <0.001		−0.60 <0.001	0.34 <0.001	−0.29 <0.01	0.18 <0.05
**WBISI**									
rho *p-value*	−0.28 <0.01	0.26 <0.01	0.28 <0.01	−0.28 <0.01	−0.60 <0.001		−0.34 <0.001	0.22 <0.05	NS
**Triglycerides**									
rho *p-value*	NS	NS	−0.20 <0.05	0.20 <0.05	0.34 <0.001	−0.34 <0.001		−0.34 <0.001	NS
**HDL–C**									
rho *p-value*	NS	0.30 <0.01	0.24 <0.01		−0.29 <0.01	0.22 <0.05	−0.34 <0.001		−0.32 <0.001
**Uric acid**									
rho *p-value*	NS	−0.40 <0.001	−0.38 <0.001	NS	0.18 <0.05	NS	NS	−0.32 <0.001	

*C, Cholesterol; FLI, Free leptin index; NS, Non-significant*.

In both sexes sOBr levels negatively correlated with all sex steroids studied. Leptin correlated negatively with testosterone levels in males and positively in females, as did with the rest of the steroids measured. Likewise, in females FLI showed positive correlations with the three androgens measured (Table 4).

Total adiponectin levels negatively correlated with androstendione in both sexes and with DHEA-S and testosterone in females, leading to a significant positive correlation between the three measured androgens and LEP/ADPr in females. HMW-adiponectin showed a negative correlation with estradiol in males (Table 4).

### Liver Steatosis

**Figure 1:**
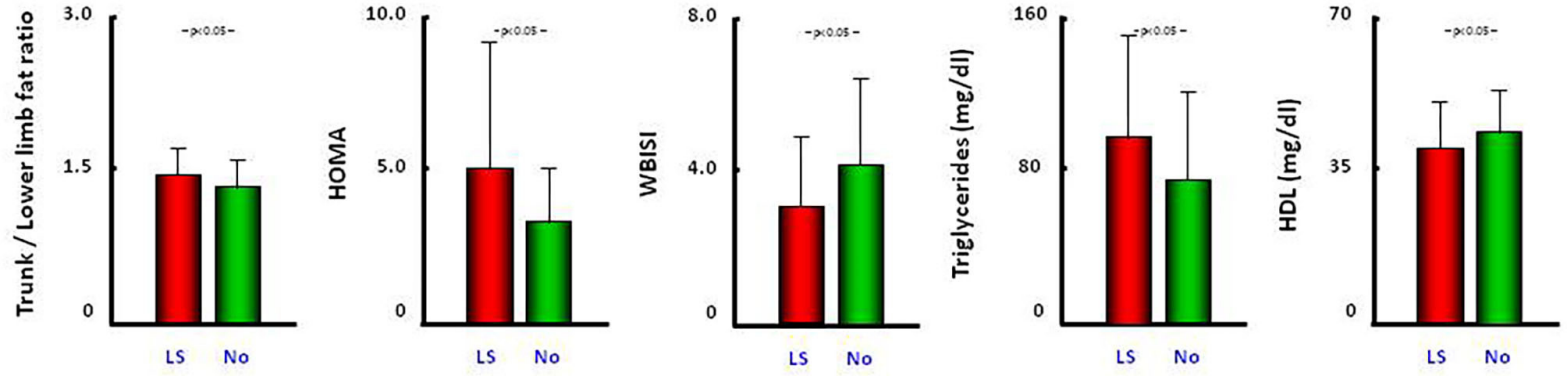
Comparison of trunk/lower limb fat ratio, HOMA, WBISI, triglycerides, and HDL between patients with liver steatosis (red bars) and without liver steatosis (green bars) status. All data are reported as the mean ± SD. LS, liver steatosis.

Liver steatosis prevalence was higher in Latinos (χ^2^: 8.69; *p* < 0.01) both in prepubertal children (33.3 vs. 16%) and adolescents (31.4 vs. 8%), with an OR of 6.76 in the logistic regression model (*p* < 0.01, Table 2). Patients with liver steatosis showed a higher Trunk/LL-fat-ratio (*p* < 0.05), higher HOMA and triglycerides (*p* < 0.05), lower WBISI (*p* < 0.01), and HDL (*p* < 0.05) that their counterparts without liver steatosis (Figure 1).

## Discussion

We have shown how body fat content, its anatomical distribution and ectopic liver deposition in childhood and adolescents with obesity is highly influenced by ethnic background, with Latino patients showing a higher rate of liver steatosis and truncal obesity, regardless of pubertal developmental status or sex despite that these two factors are known to play a relevant role in body fat distribution. Fat mass gain in adolescent obesity appears to be preferentially due to subcutaneous deposition, with pubertal adolescents showing a lower VIS/SQ-fat-ratio than prepubertal children, except in the case of pubertal Latino females. Although central adiposity in children and adolescents is metabolically important, its effects differ somewhat from the deleterious effect of visceral abdominal fat postulated for adults. Indeed, our results emphasize the relevance of the Trunk/LL-fat-ratio, which is strongly influenced by ethnicity (whereas VIS/SQ-fat-ratio is not), and its relationship with liver steatosis, insulin resistance and dyslipidemia. Moreover, we show that the correlations of sex steroids with circulating adipokine levels during pubertal development are not altered by obesity, with the exception of serum adiponectin levels where obesity blunts the effects of puberty and sex on this adipokine.

The larger amount of trunk fat in Latinos, as estimated by the Trunk/lower limb-fat-ratio, compared with Caucasians despite a similar BMI-SDS and fat%-SDS, in conjunction with the lack of effect of puberty or sex and no correlation of this ratio with sex steroids, suggests that ethnicity is perhaps the main determinant of the greater trunk fat accumulation in Latinos with obesity from early childhood. Previous observations, predominantly in Caucasian patients, have demonstrated sex differences in trunk fat adjusted for extremity fat from late puberty onwards (8), but with differences between races in the sex differences and sex-specific fat distribution associated to the progression of puberty (10). Our results support the predominant role of ethnicity, over sex and puberty, on the overall body fat distribution. It is of note that the Latino patients in our cohort were from a Peruvian, Ecuadorian or Bolivian origin, which differs from most previously published Latino cohorts, that are mainly of a Mexican-American origin. Our specific Latino population exhibits some phenotypic singularities regarding their physique as they are generally shorter and have a more stocky build compared to Caucasians.

In contrast with the Trunk/lower limb fat ratio, the VIS/SQ-fat-ratio was only found to be significantly higher in pubertal Latino females compared to Caucasian female adolescents, whereas there was not an overall higher visceral fat accumulation in Latinos. This is in general agreement with previously published differences between races regarding sex differences and sex-specific fat distribution with progression of puberty (10). However, it raises the possibility that this specific population of Latino females with obesity has singularities in fat distribution as puberty progresses.

The positive correlations found between Trunk/LL-fat-ratio and HOMA and triglycerides and the negative correlations with adiponectin and HDL, as well as the higher Trunk/LL-fat-ratio showed by patients with liver steatosis, strongly indicate the pathophysiological relevance of total trunk fat, and not only visceral fat, as stressed in adults (32), in pediatric obesity. In a large cohort of 1,300 patients with obesity (33), we demonstrated how Latino patients have higher insulin resistance markers than Caucasians in both sexes and every age range, this was endorsed in the present study by the significant effect of ethnicity on HOMA and the lower OR for WBISI observed in Latinos. The higher Trunk/LL-fat-ratio observed in Latino patients, which positively correlated with HOMA, could at least partly underlie the greater degree of insulin resistance observed in Latino vs. Caucasian children with obesity. This observation agrees with previous reports of an inverse correlation between lower limb fat content and insulin resistance (9). The negative correlation between Trunk-LL-fat ratio and total and HMW adiponectin levels, which is known to play an insulin sensitizing role, is a possible mechanistic explanation for the association between excess global trunk fat and insulin resistance. Additionally, it is of note that the Trunk-LL-fat ratio was the only body composition index showing correlation with triglyceride levels, that are known to be higher upon the existence of insulin resistance. This could also explain why the Trunk/LL-fat-ratio was the body composition parameter showing the greatest number of correlations with the studied adipokines.

Previous reports in children and adolescents have shown inter-ethnic differences in VIS/SQ-fat-ratio, consistently showing a singularly lower rate of visceral fat accumulation in African Americans regarding other ethnicities (2, 9) in contrast to a higher accumulation of visceral fat and ectopic fat deposition observed in Latinos (2). This raises the so called “ethnic paradox,” with both groups (African-Americans and Latinos) being at higher risk of metabolic derangement despite their differences in visceral fat accumulation. In our cohort, uric acid was the only metabolic parameter correlated with the VIS/SQ-fat-ratio. This finding could reinforce the lack of correlation between visceral fat content and metabolic derangement in childhood obesity, unlike adults, as well as the postulated role of uric acid as a potential marker of increased risk to develop further metabolic disturbances (34) and in raising the possibility of its use as an indirect marker of visceral fat content.

Another relevant difference observed between Latinos and Caucasians in fat deposition was the higher prevalence of liver steatosis observed in the former, which is in agreement with previous observations (12, 13, 35) and is suggested to be related with dietary habits (particularly sugar consumption) and underlying genetic predisposition in Latino patients (14, 15). The greater increase in Trunk/LL-fat-ratio in patients with liver steatosis, supports previous observations (36) indicating a role of increase trunk fat in determining ectopic fat deposition and lipotoxicity (37) that leads to a higher degree of metabolic derangement. This agrees with the higher HOMA and triglycerides and lower WBISI and HDL levels observed in the subgroup of patients affected with liver steatosis.

Pubertal Latino females are a subgroup of interest, as they showed both a higher Trunk/LL-fat-ratio and a higher VIS/SQ-fat-ratio than Caucasian female adolescents, despite having a similar BMI-SDS and fat%-SDS. They were also found to have lower leptin levels and a higher HOMA index. This set of data suggests that adolescent Latino females are at a higher risk of metabolic derangement due to singularities in their body composition and adipokine profile.

A novel finding of this study is that the VIS/SQ-fat-ratio was higher in prepubertal patients of both sexes, except for Latino females, which suggests that during adolescence the obesity associated body fat deposition occurs preferentially in the subcutaneous compartment in both sexes. In contrast, previous reports suggest that pubertal onset and progression results in sex specific differences in both overall body fat (more “android” in males) and abdominal fat (higher visceral accumulation in males) distribution (2). However, these changes are mainly observed in late puberty or early adulthood and this could be the reason why they are not observed in the pubertal patients in our cohort.

During physiological puberty males gain greater amounts of lean mass, whereas females acquire significantly more fat mass (38). Indeed, we found a negative correlation between testosterone levels and the fat/lean-ratio and leptin levels in males. Conversely, the increased tendency toward subcutaneous fat deposition in pubertal females could explain the negative correlations observed between sex steroids and visceral fat ratio in females found in this as in previous studies (39), as well as their sex specific higher leptin levels regarding prepubertal females.

In the study of adipokine profile, it is of note that although sOBr levels are already decreased in prepubertal children with obesity regarding lean children, a further decrease was observed in pubertal stages. Indeed, sOBr levels were negatively correlated with all four studied sex steroids in both sexes, as previously described in healthy children (23), suggesting that obesity did not impede the ability of this factor to respond to pubertal changes in sex steroids. In contrast, the sex and puberty induced changes in adiponectin levels previously reported in healthy subjects (23) were not observed in either total or HMW-adiponectin levels in the children and adolescents with obesity studied here.

The positive correlation of leptin with testosterone and the negative correlation of adiponectin with the three studied androgens was found only in females and mimics the hormonal and metabolic interplay described in polycystic ovary syndrome (40). The lack of correlation between androgens and adiponectin in males could be due to the decrease in this adipokine induced by both obesity and puberty in this sex (23). The negative correlation observed between HMW-adiponectin and estradiol in males could indicate an inhibitory effect of aromatization derived estradiol on adiponectin expression and multimerization (41).

The combination between sex-specific differences in fat deposition and different adipokine secretion profiles of adipose tissue according to its location (e.g., higher leptin secretion in subcutaneous *vs*. visceral adipose tissue) explains the positive correlation of FLI with fat/lean-ratio and its negative correlation with visceral fat % in the whole group (32).

The limitations of this study include the lack of control of some variables that could influence body fat content and metabolic derangement, such as the precise dietary intake of each patient, although we did confirm the lack of differences between groups in the weekly sweetened beverage consumption, or eventual differences in socio-economic status between the families based on ethnicity. Additionally, the size of the study subgroups when categorized for ethnicity, sex and pubertal status could limit the ability to find differences in the some of the studied variables. In contrast, the major strengths of this study are the exact distribution of these groups according to sex and puberty in each ethnicity and the synchronous performance of multiple image studies (ultrasonography, DXA, and MRI) and analytical determinations (metabolic parameters including OGTT, adipokine profile and sex steroids) in every patient. Additionally, Latino patients had a common geographic familial origin (most Peruvian, Ecuadorian and Bolivian) that differs from most previously published Latino cohorts (mainly from a Mexican-American origin) and who have a singular body build.

In conclusion, our results show that ethnic background strongly influences the amount of body fat and even more importantly its anatomical distribution, overwhelming the effect of sex and pubertal development regarding trunk and ectopic (liver) but not visceral fat accumulation and determining a singular predisposition to metabolic derangement (particularly insulin resistance and hypertriglyceridemia). Additionally, we have shown how the changes in body fat and distribution caused by childhood and adolescent obesity disturb the normal circulating adipokine profile in a singular way and are related to the development of metabolic derangement in childhood obesity. The trunk/lower limb fat ratio arises as a better body fat distribution index than abdominal visceral/subcutaneous fat index of insulin resistance and lipid metabolism derangement in childhood and adolescent obesity, which is in contrast to the VIS/SQ-fat-ratio being accepted in adults as the best indicator of deleterious metabolic effects but shows almost no relationship with the latter in our cohort. This constitutes a novel observation and represents an important singularity of obesity in the pediatric setting compared to this disease in adulthood.

These results could be of potential clinical relevance in the identification of patients at a higher risk of metabolic derangement and liver steatosis, as well as in the selection of the best parameters of body fat distribution to predict their metabolic risk.

## Data Availability Statement

The raw data supporting the conclusions of this article will be made available by the authors, without undue reservation.

## Ethics Statement

The studies involving human participants were reviewed and approved by Ethical Committee of the Niño Jesús University Hospital. Madrid, Spain. Written informed consent to participate in this study was provided by the participants' legal guardian/next of kin.

## Author Contributions

GM-M and JA developed the study concept and design. GM-M was responsible for patient visiting and clinical work-up. VB was in charge of adipokine level measurement. SS performed abdominal MRI and liver ultrasonography studies. FH performed DXA-scan studies. GM-M, JM-V, and RG-L were involved in data acquisition and tabulation. GM-M, JC, and JA wrote the manuscript. Result analysis and interpretation was performed by all authors. All authors critically reviewed the manuscript for accuracy and intellectual content and read and approved the final version.

## Conflict of Interest

The authors declare that the research was conducted in the absence of any commercial or financial relationships that could be construed as a potential conflict of interest.

## Abbreviations

BMI: Body Mass Index
DXA: Dual energy X-ray absorption
CT: computerized tomography
MRI: Magnetic resonance imaging
HMW: high molecular weight
SDS: Standard Deviation Score
IOTF: International Obesity Task Force
OGTT: Oral Glucose Tolerance Test
sOBr: leptin soluble receptor
DHEA-S: dehydroepiandrosterone sulfate
FLI: free leptin index
LEP/ADPr: leptin/adiponectin ratio
AUC: Area under curve
HOMA: Homeostatic Model Assessment
WBISI: Whole Body Insulin Sensitivity Index
fat%: body fat content (%)
Trunk/LL-fat-ratio: trunk to lower limb fat ratio
fat/lean-ratio: fat to lean mass ratio
VIS/SQ-fat-ratio: visceral to subcutaneous fat ratio
WMA: World Medical Association
SD: standard deviation
SPSS: Statiscal Package for Social Sciences
HDL: High Density Lipoprotein.
